# Outcomes of patients with sepsis due extensively drug-resistant bacterial infections with and without polyspecific intravenous immunoglobulin therapy: A retrospective study

**DOI:** 10.1097/MD.0000000000042190

**Published:** 2025-04-18

**Authors:** Caner Acar, Sukriye Miray Kilincer Bozgul, Haydar Cagatay Yuksel, Devrim Bozkurt

**Affiliations:** a Department of Internal Medicine, Division of Medical Oncology, Faculty of Medicine, Ege University, İzmir, Turkey; b Department of Internal Medicine, Faculty of Medicine, Ege University, İzmir, Turkey.

**Keywords:** drug resistance mortality, IVIG, septic shock, XDR pathogen

## Abstract

Sepsis caused by extensively drug-resistant (XDR) pathogens is characterized by high mortality rates. Polyspecific intravenous immunoglobulin (IVIG) has been used as an adjunctive therapy in sepsis for a long time, but it is not routinely recommended due inconclusive results. This retrospective study investigates the effect of IVIG therapy on 30-day mortality in 50 patients with sepsis caused by XDR pathogens, according to Sepsis-3 criteria. Fifty patients were included, with 28 receiving IVIG alongside standard treatment. Mortality was 74%, with no significant difference in 30-day mortality (71.4% for IVIG-treated vs 77.3% for non-IVIG-treated, *P* = .886) or intensive care unit (ICU) stay duration (median of 9.0 days for both groups, *P* = .883) between the groups. The study concludes that adding polyspecific IVIG to conventional sepsis treatment does not reduce 30-day mortality or ICU stay in XDR pathogen-induced sepsis.

## 1. Introduction

Sepsis is an organ dysfunction that develops because of the host’s uncontrolled immune response to infection.^[1]^ Following early diagnosis, resuscitation and broad-spectrum antibiotic therapy constitute the cornerstone of treatment.^[2]^ However, despite advances in supportive therapies, mortality due to septic shock remains around 40%.^[3]^ Standardized definitions for multidrug-resistant (MDR), extensive drug-resistant (XDR), and pandrug-resistant (PDR) were proposed in 2012. XDR pathogens are those that exhibit in vitro resistance to at least 1 agent in all but 2 or fewer antibiotic categories.^[4]^ These pathogens are causative agents of hospital-associated infections and are more frequently observed in patients with immune paralysis.^[5]^ The prevalence of XDR Pseudomonas aeruginosa among nosocomial pathogens ranges between 9% and 11.2%, according to the INFORM database.^[6]^ In sepsis patients infected with these pathogens, mortality rates are higher. The mortality rate in patients with infections caused by XDR Acinetobacter has been identified as 70%, whereas in the same study, the mortality rate for infections with susceptible Acinetobacter was found to be 25%.^[7,8]^ Advanced age, immunosuppressive treatment, diabetes, end-stage liver disease, steroid use, organ transplantation, and recent antibiotic use are host-associated risk factors for XDR infections.^[9]^ The positive effect of early targeted antibiotic therapy in sepsis on survival is well known.^[10]^ Because of high initial antibitoic inappropriateness and underlying immune dysfunction, treatment becomes more challenging in infections caused by XDR pathogens. Therefore, additional treatments are required for sepsis caused by XDR pathogens.^[11]^

Intravenous immunoglobulin (IVIG) therapy, due to its antibacterial and immunomodulatory effects, has long been tested as an adjunct therapy in sepsis.^[12,13]^ Although recent meta-analyses suggest its effectiveness in sepsis, it is not routinely recommended by international guidelines due to heterogeneous results.^[2]^ This study aimed to evaluate the contribution of polyspecific IVIG therapy on 30-day mortality in patients with XDR bacterial infections.

## 2. Materials and methods

### 2.1. Patient selection and study design

This retrospective study was conducted in a single-center internal medicine (non-surgical) intensive care unit (ICU) between September 2013 and February 2021. The Institutional Ethical Review Board of Ege University Hospital approved the study (21-6.1T/54). The study was conducted in accordance with good clinical practice guidelines and adhered to the principles of the Declaration of Helsinki. Patients or their relatives provided written informed consent. We included all adult patients (≥18 years old) diagnosed with sepsis due to XDR bacterial infection.

### 2.2. Data collection

Following data were retrieved using electronic medical records: age, sex, length of ICU stay, comorbidities, site of infection, isolated pathogens and inflammatory markers among laboratory parameters. The sequential organ failure assessment score (SOFA) on admission were calculated. During ICU stay; presence of organ dysfunction, vasopressor support (norepinephrine), need for renal replacement therapy, glasgow coma score change, appropriateness of empiric antimicrobials on the first day and 30-day mortality were recorded. Source of infection was hospital-acquired in all patients. Isolated pathogens were also recorded.

The diagnosis of sepsis and septic shock were made according to the Sepsis-3 criteria.^[1]^ The definition of XDR pathogen was defined, according to the international guideline recommendation, as non-susceptible to ≥ 1 agent in all but ≤ 2 antibiotic categories.^[4]^ Empirical antibiotic therapy was considered inappropriate if the isolate did not display in vitro susceptibility to any systemic antibiotic administered on the day of culture sampling. Standard sepsis treatment was administered to all patients (initiation or modification of broad-spectrum antibiotic therapy, IV fluid resuscitation, vasopressors, hemodialysis and ventilator support as needed). Glucocorticoid therapy was not used for sepsis treatment. The standard dose for polyspecific IVIG therapy was 1 g/kg over 2 days and initiated within the first 48 hours after sepsis diagnosis. IVIG treatment was administered based on its availability in the hospital during the period of treatment. The clinical conditions of the patients did not influence the decision for treatment.

**Figure 1.**
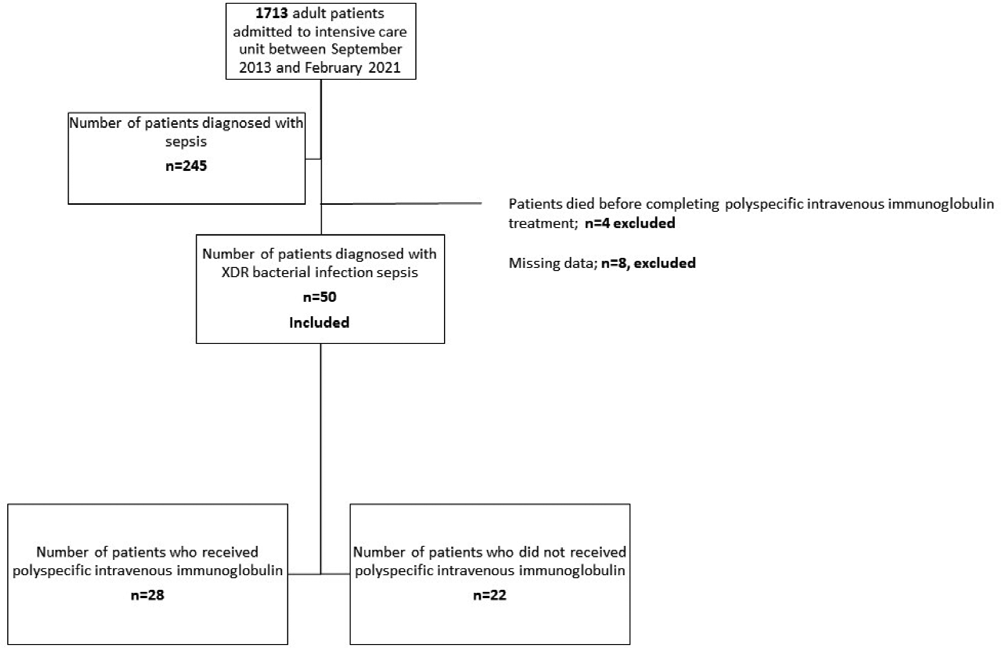
Flowchart of patient inclusion.

Patients under 18 years of age, those who died before completing IVIG treatment, and patients with incomplete data for the evaluation of sepsis-3 criteria were excluded. Figure 1 shows the patient inclusion. Primary outcome was 30-day mortality and the secondary outcome was ICU length of stay among patients who received polyspecific IVIG therapy and those who did not.

### 2.3. Statistical analysis

Descriptive statistics were used to summarize the data obtained from the study. Continuous (numerical) variables are presented in tables as mean ± standard deviation or median, minimum, and maximum, depending on the distribution. Categorical variables are summarized as counts and percentages. The normality of the numerical variables was checked using Shapiro–Wilk, Kolmogorov–Smirnov, and Anderson–Darling tests. In statistical analyses, a significance level of *P* < .05 was used and considered 2-sided to evaluate differences in both directions.

For comparisons between 2 independent groups, the Independent Samples *t*-test was used when numerical variables were normally distributed, whereas the Mann–Whitney *U* test was used when they were not normally distributed. Pearson chi-square test was used for comparisons of differences between categorical variables in groups in 2 × 2 tables where expected counts were 5 or more, Fisher exact test was used for tables where expected counts were <5, and Fisher Freeman Halton test was used for R C tables where expected counts were <5. Additionally, a multivariate regression analysis was performed to evaluate the impact of IVIG on 30-day mortality. Missing values were not addressed or imputed and were thus excluded from analyses. Statistical analyses were conducted using “Jamovi project (2020), Jamovi (Version 1.8.4.0) [Computer Software]” (retrieved from https://www.jamovi.org) and JASP (Version 0.14.1.0) (retrieved from https://jasp-stats.org) programs, and a significance level of .05 (*P*-value) was considered for statistical analyses.

## 3. Results

During the study period, 245 patients diagnosed with sepsis were followed up in the ICU. Among them; in 50 patients (20.4%), XDR pathogens were identified as the causative agents of sepsis. Demographic information, comorbid conditions, presence of organ failure, and infection site of these patients, along with the differences between the 2 groups, are presented in Table 1. The median age of the group receiving IVIG therapy was 55 years, whereas it was 63 years those who did not received IVIG (*P* = .024). No significant difference was found between the 2 groups in terms of gender and comorbid conditions (*P* > .05 for each). In the assessment of the severity of sepsis between the 2 groups, the median SOFA score was 9.5 [3–11] in the group not receiving IVIG and 9 [3–15] among the patients who received IVIG (*P* = .478). There was no difference between the 2 groups in the SOFA scores and organ failure assessments (*P* > .05 for each). Additionally, there was no difference in the sources of infection causing sepsis and the rates of appropriate initial antibiotic therapy use (40.9% vs 42.9%, respectively, *P* = .999). In the 30-day mortality analysis, mortality was found to be 71.4% in the group receiving IVIG therapy and 77.3% in the group not receiving IVIG therapy (*P* = .886). The impact of IVIG use on 30-day mortality was evaluated after adjusting for age, SOFA score, appropriate antibiotic use, infection site, and vasopressor need. In this multivariate regression analysis, the adjusted odds ratio for IVIG use was 0.867 (95% CI: 0.126–5.968, *P* = .885). The length of ICU stay (days) in the group receiving IVIG therapy was a median of 9.0 [3.0–58.0] days, and in the group not receiving IVIG therapy, it was a median of 9.0 [2.0–48.0] days (*P* = .883).

**Table 1:** Demographic and clinical characteristics of patients.

	IVIG		*P*
	No (n = 22)	Yes (n = 28)	
Age*	63.0 [20.0–86.0]	55.0 [18.0–76.0]	.024
Gender†			
Male	9 (40.9)	18 (64.3)	.174
Female	13 (59.1)	10 (35.7)	
Length of ICU stay, d*	9.0 [2.0–48.0]	9.0 [3.0–58.0]	.883
Mortality, present†	17 (77.3)	20 (71.4)	.886
Comorbidity†	16 (72.7)	17 (60.7)	.556
Diabetes mellitus	9 (40.9)	7 (25.0)	.373
Cardiovascular disease	5 (22.7)	7 (25.0)	.999
Chronic heart failure	2 (9.1)	4 (14.3)	.683
COPD/asthma	5 (22.7)	1 (3.6)	.075
Chronic renal disease	2 (9.1)	4 (14.3)	.683
Vasculitis	1 (4.5)	3 (10.7)	.621
Connective tissue disease	1 (4.5)	2 (7.1)	.999
Hematological malignancy	6 (27.3)	12 (42.9)	.399
Solid organ malignancy	0 (0.0)	1 (3.6)	.999
Site of infection†			
Pneumonia	10 (45.5)	13 (46.4)	.999
Urinary tract	6 (27.3)	4 (14.3)	.302
Catheter and blood stream	4 (18.2)	5 (17.9)	.999
Abdomen (other than biliary system)	1 (4.5)	3 (10.7)	.621
Biliary system	1 (4.5)	1 (3.6)	.999
Skin and soft tissue	2 (9.1)	2 (7.1)	.999
Meningitis	0 (0.0)	1 (3.6)	.999
Septic arthritis	1 (4.5)	0 (0.0)	.440
Primary bacteremia (source can’t find)	0 (0.0)	1 (3.6)	.999
Organ dysfunction			
Heart rate*	114.0 [95.0–145.0]	117.0 [79.0–160.0]	.971
Hypotension, yes†	20 (90.9)	22 (78.6)	.439
Vasopressor support, yes†	18 (81.8)	17 (60.7)	.106
Acute kidney injury, yes†	15 (68.2)	14 (50.0)	.315
Renal replacement therapy, yes†	7 (31.8)	9 (32.1)	.999
Hepatobiliary dysfunction, yes†	3 (13.6)	8 (28.6)	.306
Disseminated intravascular coagulation, yes†	4 (18.2)	7 (25.0)	.734
Thrombocytopenia (<150,000 µL), yes†	15 (68.2)	23 (82.1)	.416
Acute respiratory failure, yes†	11 (50.0)	14 (50.0)	.999
Glasgow coma score change, *yes*†	6 (27.3)	9 (32.1)	.950
SOFA score*	9.5 [3–11]	9 [3–15]	.478
Appropriateness of empiric antimicrobials on day 1†	9 (40.9)	12 (42.9)	.999

Abbreviations: COPD = chronic obstructive pulmonary disease, ICU = intensive care unit, IVIG = intravenous immunoglobulin, SOFA = sequential organ failure assessment.

* Median [Min. to Max.].

† n (%).

The laboratory results at the time of sepsis diagnosis and isolated pathogens are shown in Table 2. No difference was found between the 2 groups in terms of inflammatory markers and lactate levels. Upon examining the culture results, it was observed that in a total of 49/50 patients, gram-negative XDR pathogens were isolated, and in only 1 patient in the group not receiving IVIG, a gram-positive XDR bacteria was the causative agent of sepsis. The other culture isolates are listed in Table 2. There was no statistically significant difference between those who received and did not receive IVIG in terms of the proportions of pathogens identified in the cultures (*P* > .05 for each).

**Table 2:** Hematological, biochemical parameters and microbiological findings of patients.

	IVIG		*p*
	No (n = 22)	Yes (n = 28)	
Neutrophil count, /µL*	4085.0 [0.0–19700.0]	1500.0 [0.0–57930.0]	.310*
Lymphocyte count, /µL*	450.0 [0.0–5840.0]	240.0 [0.0–3660.0]	.100*
Albumin, g/dL*	2.8 [1.8–4.3]	2.5 [1.8–3.5]	.429*
Lactate dehydrogenase, U/L*	288.0 [155.0–1409.0]	295.0 [99.0–927.0]	.615*
Lactate, mmol/L*	2.8 [0.6–9.4]	3.1 [1.3–7.2]	.463*
C-reactive protein, (mg/L)*	225.5 [53.0–329.0]	235.0 [32.0–366.0]	.333*
Procalcitonin, µg/L*	3.0 [0.3–50.0]	7.3 [2.2–100.0]	.064*
Isolated pathogens†			
ESBL producing Enterobacteriaceae	1 (4.5)	2 (7.1)	.999
Carbapenem-resistant Enterobacteriaceae	6 (27.3)	14 (50.0)	.181
Methicillin-resistant *Staphylococcus Aureus*	1 (4.5)	0 (0.0)	.440
*Pseudomonas* spp.	9 (40.9)	4 (14.3)	.071
* Acinetobacter baumannii*	10 (45.5)	12 (42.9)	.999
Methicillin-resistant CoNS	2 (9.1)	1 (3.6)	.576
*Candida* spp.	1 (4.5)	1 (3.6)	.999
Cytomegalovirus infection	0 (0.0)	2 (7.1)	.497
*Aspergillus* spp.	0 (0.0)	1 (3.6)	.999
* Enterococcus faecalis*	4 (18.2)	2 (7.1)	.385
ESBL negative enterobacteriaceae	1 (4.5)	0 (0.0)	.440
Methicillin-sensitive *Staphylococcus Aureus*	1 (4.5)	0 (0.0)	.440
Other	1 (4.5)	1 (3.6)	.999
Gram-negative bacteria isolation†	21 (95.5)	28 (100.0)	.440
Gram-positive bacteria isolation†	7 (31.8)	3 (10.7)	.084
Bacteremia†	16 (72.7)	19 (67.9)	.950

Abbreviations: CoNS = coagulase-negative staphylococci, ESBL = extended-spectrum beta-lactamase, IVIG = intravenous immunoglobulin.

* Median [Min. to Max.].

† n (%).

## 4. Discussion

Recent studies have shown that during sepsis, pro-inflammatory and anti-inflammatory processes are activated simultaneously. Following the recognition of Pathogen-associated molecular patterns and Damage-associated molecular pattern molecules by the immune system, the release of cytokines and mediators occurs, leading to a cytokine storm. In addition, T-cell exhaustion and apoptosis in immune cells result in immunosuppression, which increases the risk of nosocomial infections, prolongs hospital stays, and can lead to death. This double-edged inflammatory process in sepsis poses challenges in the application of immunomodulatory treatments.^[14]^

Patients with sepsis caused by XDR pathogens tend to have higher mortality rates than those caused by other pathogens.^[15]^ The high mortality rate in these patients is generally due to the prevalent immune dysfunction and ineffectiveness of empirical antibiotics.^[16]^ Studies to enhance the effectiveness of antibiotic therapy using new antibiotics and combined regimens are ongoing.^[17,18]^ However, failure or delay in pathogen isolation results in patients being deprived of effective antibiotic therapy during this period.^[11]^ During this time, when they are not receiving effective antibiotics, XDR pathogens can induce a higher inflammatory response than susceptible pathogens, leading to septic shock.^[19]^ To suppress this inflammatory response, methods such as extracorporeal blood purification techniques and IVIG therapy can be used in patients with sepsis caused by XDR pathogens.^[20,21]^

IVIG contains a broad spectrum of antibodies obtained from live donors.^[22]^ These antibodies have antibacterial effectiveness against microorganisms.^[23]^ In vitro studies have shown that adding IVIG along with antimicrobial agents to *Acinetobacter baumannii* isolates can increase bacterial cell damage.^[24]^ In addition to its antibacterial efficacy, it has an immunomodulatory effect on the excessive inflammatory response developed against the pathogen.^[25]^ It can also contribute to the prevention of secondary infections that may arise due to potential immune paralysis during sepsis.^[26]^ Due to its antibacterial, immunomodulatory effects and potential to replenish decreased Ig levels in sepsis, IVIG can reduce both early mortality related to the high inflammatory condition in the initial phase of sepsis and long-term hospitalization-related mortality due to the late phase low-grade inflammatory, immunosuppressed phenotype. Therefore, IVIG therapy is an attractive treatment option, especially in patients with sepsis and resistant microorganisms.

Studies indicate that the timing of administration (early or late), type of IVIG preparation (IgM-enriched or standard IVIG), and the dose of IVIG can influence its effectiveness. Evidence suggests that use within the first 24 hours, high-dose IVIG therapy (>1 g/kg), and IgM-enriched preparations may be more effective.^[27,28]^ However, due to the heterogeneous results in studies, it is not routinely recommended in the current guidelines.^[2]^

There are limited data in the literature regarding the effectiveness of IVIG therapy in MDR and XDR pathogens. In a previous study evaluating IVIG therapy in sepsis patients with MDR and XDR pathogens, the positive effect of IgM-enriched IVIG therapy on survival was demonstrated. However, there are no direct data on the effectiveness of standard IVIG therapy in this patient group.^[29]^

In our retrospective study evaluating the effectiveness of IVIG therapy in 50 patients with sepsis caused by XDR bacteria, there was no difference between the group that received IVIG therapy and the group that did not in terms of sepsis severity and inflammatory markers. Additionally, both groups were equal in terms of using effective antibiotic therapy initially. In the 2 groups with similar baseline characteristics, no difference was observed in 30-day mortality and ICU stay durations with IVIG treatment. Based on these results, it has been concluded that standard IVIG therapy is not effective in patients with sepsis caused by XDR bacteria.

Recent meta-analyses have suggested that IgM-enriched IVIG therapy in sepsis treatment may be more effective than standard IVIG therapy.^[30]^ The pentameric structure of IgM enhances its antimicrobial and opsonization activities. Therefore, it may strengthen standard treatment, especially in patients with gram-negative infections who are not receiving effective antibiotic therapy.^[12]^ The IVIG preparations used in our patients were all IgM poor, which might be the reason for the inability to demonstrate a difference in survival.

We presented a highly selected patients group, however, the study’s retrospective, single-center nature and the inclusion of a small number of patients (n = 50) are limitations and have limited generalizability. However, despite its retrospective nature, there was no significant difference in SOFA scores among the included patients, indicating 2 comparable groups of patients in terms of sepsis severity, is the major strength of our study. All patients used the same IVIG preparation, and the study did not include heterogeneity in terms of administration dose (1 g/kg over 2 days) and the initiation time (within the first 48 hours).

## 5. Conclusion

Mortality rates are high in XDR pathogen related sepsis and septic shock. We noted that in the similar severity illness populations, contribution of polyspecific IVIG on standard therapy does not appear have effect on 30-day mortality and ICU length of stay. There is limited research on the effectiveness of polyspecific IVIG therapy in patients with sepsis caused by resistant pathogens. IVIG therapy with IgM-enriched preparations might be more appropriate particularly in hospital-acquired sepsis, where there is a risk of resistant pathogens and doubts about the adequacy of empirical antibiotic treatment.

## Author contributions

**Conceptualization:** Caner Acar, Devrim Bozkurt.

**Data curation:** Caner Acar, Sukriye Miray Kilincer Bozgul, Haydar Cagatay Yuksel.

**Formal analysis:** Caner Acar, Sukriye Miray Kilincer Bozgul.

**Investigation:** Caner Acar, Haydar Cagatay Yuksel.

**Methodology:** Caner Acar.

**Project administration:** Caner Acar.
